# Plasma Total Tau and Neurobehavioral Symptoms of Cognitive Decline in Cognitively Normal Older Adults

**DOI:** 10.3389/fpsyg.2021.774049

**Published:** 2021-11-05

**Authors:** James R. Hall, Melissa Petersen, Leigh Johnson, Sid E. O’Bryant

**Affiliations:** ^1^Institute for Translational Research, University of North Texas Health Science Center, Fort Worth, TX, United States; ^2^Department of Pharmacology and Neuroscience, University of North Texas Health Science Center, Fort Worth, TX, United States; ^3^Department of Family Medicine, University of North Texas Health Science Center, Fort Worth, TX, United States

**Keywords:** plasma tau, depression, apathy, anxiety, worry, daytime sleepiness, cognitively normal, older adults

## Abstract

Depression and related neurobehavioral symptoms are common features of Alzheimer’s disease and other dementias. The presence of these potentially modifiable neurobehavioral symptoms in cognitively intact older adults may represent an early indication of pathophysiological processes in the brain. Tau pathology is a key feature of a number of dementias. A number of studies have found an association between tau and neurobehavioral symptoms. The current study investigated the relationship of a blood-based biomarker of tau and symptoms of depression, anxiety, worry, and sleep disturbances in 538 community based, cognitively normal older adults. Logistic regression revealed no significant relationship between plasma total tau and any measures of neurobehavioral symptoms. To assess the impact of level of tau on these relationships, participants were divided into those in the highest quintile of tau and those in the lower four quintiles. Regression analyses showed a significant relationship between level of plasma total tau and measures of depression, apathy, anxiety, worry and sleep. The presence of higher levels of plasma tau and elevated neurobehavioral symptoms may be an early indicator of cognitive decline and prodromal Alzheimer’s disease. Longitudinal research is needed to evaluate the impact of these factors on the development of dementia and may suggest areas for early intervention.

## Introduction

A number of neurobehavioral symptoms including depression, apathy, anxiety (Ma, 2020), worry (Bower et al., 2019), and sleep disturbances (Ju et al., 2014) have been shown to be risk factors for the development of cognitive decline. A history of depression (Geda et al., 2014) as well as the occurrence of late life depression (Linnemann and Lang, 2020) have been linked to the development of Alzheimer’s disease (AD). Depression has been found to be both a risk factor for cognitive decline (Diniz et al., 2013) as well as a preclinical symptom of AD (Donovan et al., 2014; Kuo et al., 2020). Depression is among the most frequent neuropsychiatric disorders accompanying AD and related dementias (Lyketsos et al., 2011; Zhao et al., 2016). Burke et al. (2018) using the National Alzheimer’s Coordinating Center data from 12,083 cognitively normal participants found that depression, anxiety and sleep disturbances were associated with the risk of AD.

Symptoms of apathy have been associated with increased dementia risk in a community dwelling cohort and suggested to be prodromal to the development of dementia (van Dalen et al., 2018). Apathy was found to be a prodromal symptom of dementia in small vessel disease (Tay et al., 2020). Anxiety has been shown to be a predictor of cognitive decline and dementia (Gulpers et al., 2016). Santabárbara et al. (2020) in a meta-analysis of prospective cohorts with 29,608 participants concluded that anxiety was significantly associated with all-cause dementia. Worry has been shown to be a predictor of decline in learning and memory in cognitively normal older adults (Pietrzak et al., 2012). Worry is considered a modifiable predictor of cognitive decline and dementia (Bower et al., 2019). In a study of the association of worry, anxiety and depression to cognitive performance in a sample of older adults, worry was found to have a significantly greater negative impact than either anxiety or depression (de Vito et al., 2019). Sleep disturbances have been related to AD as either a marker of or a mechanism mediating the risk for AD (Lucey, 2020). Short sleep duration (Spira et al., 2013), increased napping (Owusu et al., 2019) and excessive daytime sleepiness have all been found to be predictive of cognitive decline (Keage et al., 2012; Carvalho et al., 2018). A recent meta-analysis found that depression and sleep duration (long or short) were the symptoms most consistently associated with cognitive decline (Hudon et al., 2020).

The presence of these potentially modifiable neurobehavioral symptoms in cognitively intact older adults may represent an early indication of pathophysiological processes in the brain. The Amyloid, Tau and Neurodegeneration [AT(N)] biologically based framework for understanding AD (Jack et al., 2018) emphasizes the role of markers of amyloid accumulation, tau and neurodegeneration. Although there have been inconsistent results relating affective symptoms to AD biomarkers (Banning et al., 2019) there is suggestive evidence that supports this line of inquiry.

Tau is a brain specific microtubule associated protein. The intracellular aggregation of tau produces neurofibrillary tangles, which are a primary feature of AD neuropathology (Iqbal et al., 2010). Tau abnormalities related to hyperphosphorylation have been found in over 20 neurodegenerative brain disorders including Parkinson’s dementia, Lewy Body Dementia and Corticobasal Degeneration (Kovacs, 2017). Although the exact nature of the role of tau in affective symptoms is unclear, a number of studies have found an association between tau and neurobehavioral symptoms. A study of memory clinic patients found a significant correlation between level of tau and behavioral and psychological symptoms of dementia (Cotta Ramusino et al., 2021). Research on tau levels in cognitively normal older adults has shown that those with elevated tau were twice as likely to be depressed (Babulal et al., 2020). In a study of the trajectory of depression and apathy over time in prodromal Alzheimer’s, lower Aβ_42_ and higher tau were related to an increased likelihood of depression and apathy (Banning et al., 2021). Apathy has been associated with the accumulation of tau in the right frontal regions of the brain (Marshall et al., 2019). Anxiety has been related to the level of total tau (t-tau) in individuals with mild cognitive impairment (Ramakers et al., 2013). Disturbances in the sleep cycle has been found to be effected by tau accumulation in cognitively normal and those with very mild cognitive impairment (Lucey et al., 2019; Benedict et al., 2020).

The vast majority of studies on the role of tau have used CSF markers of tau or with the availability of tau radiotracers, positron emission tomography (PET) tau biomarkers. The addition of plasma tau to CSF tau has been shown to improve diagnostic accuracy (Fossati et al., 2019). Although the association between plasma t-tau and CSF tau has been found to be relatively weak, (Mattsson et al., 2016; Pase et al., 2019) a number of studies have supported the use of plasma in investigating Alzheimer’s risk (Chiu et al., 2014; Mielke et al., 2017). Compared to other methods of assessing tau, blood based biomarkers have the added benefit of not requiring the invasiveness of a spinal tap nor the expense and lack of availability of tau PET. The current study utilizes blood-based biomarkers of t-tau to investigate the relationship between plasma total tau and symptoms of depression, apathy, anxiety, worry and sleep disturbances in a cohort of community based cognitively normal older adults.

## Materials and Methods

### Participants

Participants were drawn from the HABLE cohort a community-based, longitudinal study of cognitive aging in Mexican-Americans. A 409 cognitively normal Mexican Americans (323 females, 86 males) and 129 cognitively normal non-Hispanic whites (91 females, 38 males) made up the sample. A full description of the HAB LE protocol has been published elsewhere (O’Bryant et al., 2021). Briefly, the HAB LE study protocol includes a functional exam, blood draw, neuroimaging, clinical labs, interview (medical history, family medical history, and sociocultural factors), neuropsychological testing and the assessment of neuropsychiatric symptoms. Study material is administered in either English or Spanish per the participants reported language preference. The HAB LE protocol is conducted under IRB protocols 2016-128 and 2017-165 and all participants and/or caregivers sign written informed consent. All participants are evaluated at a single site within the Institute of Translational Research at the University of North Texas Health Science Center, Fort Worth, Texas.

### Affective Measures

Depressive symptomology was assessed using the Geriatric Depression Scale 30-item (GDS) (Yesavage et al., 1982) a scale designed to be used for screening depression in the elderly. A factor analytic study (Hall and Davis, 2010; Hall et al., 2011) revealed four factors and based on that analysis, the GDS was divided into four symptom subscales. Dysphoria (11 items) – related to sad mood; Meaninglessness (7 items) – evaluating the meaning or lack of meaning in one’s life; Apathy (6 items) – associated with absence of motivation and Cognitive Impairment (6 items) – having awareness and concern of one’s cognitive decline.

The level of anxiety was assessed using the Beck Anxiety Inventory (BAI) (Beck et al., 1988). The BAI is a 21-item state anxiety scale measuring the intensity of cognitive, affective, and somatic anxiety symptoms experienced during the last 7 days. The scale is composed of two factors: physical symptoms and cognitive symptoms of anxiety (Beck et al., 1988).

The Penn State Worry Questionnaire (PSWQ) (Meyer et al., 1990) is a 16-item questionnaire that assesses the trait of worry, using a Likert rating from 1 (not at all typical of me) to 5 (very typical of me) and measures the tendency of an individual to engage in excessive, generalized, and uncontrollable worry.

The Epworth Sleepiness Scale (ESS) (Johns, 1991) is an eight-item measure aiming to assess daytime sleepiness and is an indirect assessment of sleep difficulties that may be associated with depression and cognitive decline.

### Diagnostic Classification

Using a classification decision tree, normal cognition was assigned based on the following: (1) no complaints of cognitive change (self or other) and (2) Clinical Dementia Rating scale sum of boxes score = 0 and (3) all cognitive test scores when converted to z scores fell broadly within normal testing limits which was defined as being no greater than −1 z score.

### Blood Processing

Fasting blood collection and processing were completed based on the international guidelines for AD biomarker studies (O’Bryant et al., 2015) and processed within 2 h (stick-to-freezer). Samples were assayed in the University of North Texas Health Science Center Institute for Translational Research (ITR) Laboratory by the ITR Biomarker Core. The ITR Biomarker Core utilizes the Hamilton Robotics EasyBlood for blood processing, aliquoting and re-aliquoting. A total of 500 μl of plasma was utilized to measure biomarker levels using the Single Molecule Array (Simoa) technology (Simoa; Quanterix, Lexington, MA, United States). Tests were performed to optimize dilution factors and centrifugation and the suggested dilution factor of 4× was suitable for our samples. After thawing, the samples were vortexed and spun at 10,000 g for 5 min; the supernatant was directly transferred to a 96 well plate.

### Assaying Tau

Utilizing Simoa technology, multiplexed detection of t-tau was accomplished by labeling beads with dyes of various wavelengths and concentrations creating distinct subpopulations of beads. Antibodies for each specific protein were immobilized to these color-encoded beads. Mixture of these beads were incubated with each sample generating detection of multiple proteins. From the materials provided, a recombinant 3-Plex calibration curve was constructed and transferred to the 96 well plate. Calibration range for plasma t-tau was 0–100 pg/mL and dynamic range of 0–400 pg/mL. The t-tau control samples (analog 2.24 pg/mL) and inter-assay control (pooled normal plasma) were all transferred to the 96 well plate. The sample and control concentrations were calculated from 4 PL curve fit. CV for t-tau was reported at 0.061. LLODs for t-tau were reported at 0.019 pg/mL. Interplate CVs were derived for high and low pooled controls from the Quanterix automated system and for t-tau, High control CV = 0.040, Low control CV = 0.047.

### Statistical Analyses

Data were analyzed using SPSS-25 (IBM). Independent *t*-tests were conducted to examine differences in demographic characteristics and differences between quintile groups. Chi squared analysis was applied to categorical data. Regression models were created to examine the link between t-tau and symptoms of depression, anxiety worry and sleepiness with t-tau, age, sex and education as predictors. Regression models were created to examine the impact of level of t-tau (quintiles), age, sex and education on each of the affective measures. Statistical significant was set at *p* < 0.05.

## Results

Table 1 presents the characteristics of the sample. The two ethnic groups did not differ on plasma tau nor on any of the affective measures, hence the two groups were combined for analysis. Regression analyses (Table 2) were conducted to assess the ability of plasma total tau to predict symptoms of depression, anxiety, worry and sleep disturbances in a sample of cognitively normal older adults. Age, sex, and education along with plasma total tau were entered as predictors. Total tau was not a significant predictor of any of the affective measures.

**TABLE 1:** Characteristics of the sample.

	**Total sample**	**Lower quintiles**	**Highest quintile**
	***N* = 538**	***N* = 428**	***N* = 110**
Age	*M* = 59.941	*M* = 59.815	*M* = 60.429
	SD = 7.792	SD = 7.813	SD = 7.628
Education	*M* = 9.719	*M* = 9.644	*M* = 10.001
	SD = 4.795	SD = 4.895	SD = 4.594
Gender % Female	77%	77%	79%
Plasma total tau	*M* = −6069.746	*M* = 1.569	*M* = 3.208
	SD = 71411.513	SD = 0.441	SD = 1.347

**TABLE 2:** Linear regression for plasma total tau and tau quintiles and affective measures.

**Plasma total tau**				**Plasma tau quintiles**		
**Affective measures**	**β**	***t***	***p***	**β**	***t***	***p***
Geriatric depression scale (GDS) total	0.052	1.251	0.211	0.088	2.088	0.037*
GDS dysphoria	0.036	0.868	0.386	0.067	1.585	0.114
GDS meaninglessness	0.032	0.750	0.454	0.049	1.152	0.250
GDS apathy	0.062	1.456	0.146	0.113	2.680	0.008*
GDS cognitive impairment	0.058	1.394	0.164	0.068	1.620	0.106
Beck anxiety inventory (BAI)	−0.015	−0.344	0.731	0.090	2.128	0.034*
BAI physical symptoms	−0.003	−0.066	0.947	0.110	2.594	0.010*
BAI cognitive symptoms	−0.024	−0.550	0.583	0.098	2.289	0.022*
Penn state worry questionnaire	0.007	0.155	0.877	0.118	2.792	0.005*
Epworth sleepiness scale	−0.029	−0.684	0.494	0.100	2.326	0.020*

* ≤ 0.05.

To assess the possible impact of level of t-tau, the sample was divided into those in the highest quintile of plasma tau (*N* = 110) and those in the lower 4 quintiles of t-tau (*N* = 428). The characteristics of the sample are shown in Table 1. The quintile groups did not differ in age (*t* = −0.744, df = 536, *p* = 0.457) education (*t* = 0.467, df = 536, *p* = 0.476) nor distribution of the sexes (*X ^2^* = 0.1984, *p* = 0.656). Table 3 presents the scores on each of the affective measures comparing the two group. Those in the highest quintile scored significantly higher on GDS total score, GDS Apathy subscale, BAI total score, BAI Physical Symptoms, BAI Cognitive Symptoms, PSWQ and the ESS.

**TABLE 3:** Scores on affective measures X quintile group.

**Affective measures**	**Lower quintiles**	**Highest quintile**	***p***
	***N* = 428**	***N* = 110**	
Geriatric depression scale (GDS) total	*M* = 7.89	*M* = 9.33	*t* = −1.977 df = 536
	SD = 6.702	SD = 7.240	*p* = 0.049*
GDS dysphoria	*M* = 2.65	*M* = 3.08	*t* = −1.333 df = 536
	SD = 2.981	SD = 3.160	*p* = 0.183
GDS meaninglessness	*M* = 1.12	*M* = 1.30	*t* = −1.029 df = 536
	SD = 1.576	SD = 1.733	*p* = 0.304
GDS apathy	*M* = 1.64	*M* = 2.11	*t* = −2.879 df = 536
	SD = 1.512	SD = 1.586	*p* = 0.004*
GDS cognitive impairment	*M* = 2.49	*M* = 2.76	*t* = −1.445 df = 536
	SD = 1.752	SD = 1.733	*p* = 0.149
Beck anxiety inventory (BAI)	*M* = 5.20	*M* = 7.55	*t* = −2.5835 df = 536
	SD = 7.551	SD = 8.858	*p* = 0.010*
BAI physical symptoms	*M* = 2.82	*M* = 3.84	*p* = 0.030*
	SD = 4.231	SD = 4.881	*p* = 0.030*
BAI cognitive symptoms	*M* = 2.35	*M* = 3.36	*t* = −2.434 df = 536
	SD = 3.743	SD = 4.455	*p* = 0.015*
Penn state worry questionnaire	*M* = 39.48	*M* = 44.205	*t* = −2.779 df = 536
	SD = 15.809	SD = 16.301	*p* = 0.006*
Epworth sleepiness scale	*M* = 4.52	*M* = 5.564	*t* = −2.411 df = 536
	SD = 4.030	SD = 4.465	*p* = 0.016*

*p ≤ 0.05.

To evaluate the possible clinical significance of these differences, the two groups were compared using accepted clinical cutoff scores. Applying the clinical cutoff of 9 (Laudisio et al., 2018) to the GDS, 42% of the highest quintile group scored higher than the cutoff while 32% of those in the lower quintiles scored above the cutoff (*X*^2^ = 3.805, *p* = 0.049). On the BAI 35% of those in the highest quintile scored above the clinical cutoff of 14 (Diefenbach et al., 2009) and 24% of those in the lower quintiles scored above the cutoff (*X*^2^ = 5.032, *p* = 0.025). When the clinical cutoff of 50 was applied to the PSWQ (Wuthrich et al., 2014), 38% of those in the highest quintile scored above 50 compared to 27% of those in the lower quintiles (*X*^2^ = 4.666, *p* = 0.031). Comparing the groups on the ESS using a cutoff of 10 (Johns, 1991), 21% of those in the highest group scored above 10 and 9.7% of those in the lower quintiles were above the cutoff (*X*^2^ = 10.459, *p* = 0.001).

Regression analyses were conducted to assess the effect of level of total tau on each of the affective measures. Level of t-tau (quintile groups), age, sex and education were entered as predictors. Table 2 presents the results of the regression analyses showing that level of plasma t-tau was a significant predictor of total depression and symptoms of apathy. The scores on measures of anxiety and worry were significantly predicted by the level of t-tau, as was the score on a measure of sleep difficulty.

## Discussion

This study is one of the first to show the association of plasma t-tau levels with levels of symptoms of depression, anxiety, worry and daytime sleepiness in cognitively normal older adults. The current findings are consistent with earlier findings using CSF and PET data that t-tau is related to the presence of a range of neurobehavioral symptoms in cognitively normal older adults but only at the highest level. Our findings suggest that the association between depression and other neurobehavioral disorders and tau related AD pathology may vary depending on the type and severity of the affective symptoms and the level of peripheral tau.

There are a number of strengths and limitations to the study. The participants were community-based and a robust diagnostic algorithm was applied to determine cognitive status. The study assessed a range of neurobehavioral symptoms. The current research investigated plasma tau, a more accessible biomarker of cognitive decline than CSF tau or tau imaging. The limitations are related to the sample and methodological approach used in the study. The sample was drawn from a study of cognitive aging in Mexican-Americans and was composed predominately of Mexican-Americans, which limits the generalizability of the findings. The current study utilized cross-sectional data and the nature of changes over time and the impact of the progression of cognitive decline on the tau – affective symptom relationship could not be assessed. The analysis utilized scores on measures that are frequently used in research settings but much less so in primary care settings (Olariu et al., 2015; Bhattacharjee et al., 2018) limiting the applicability of the findings. Other factors that may mediate the tau-affective symptom relationship such as apolipoprotein-ε4 status need to be investigated. Longitudinal research with a larger sample size and more representative of the overall population would be useful.

A number of questions arise from the results of this study that can only be answered by longitudinal data. The most salient is determining if elevated plasma tau is a risk for cognitive decline when associated with symptoms of affective disorders. Specifically, which of these symptoms or combination of symptoms when paired with elevated plasma t-tau are the best predictors of the risk for Alzheimer’s and other dementias. For example, is elevated plasma t-tau combined with subsyndromal depressive symptoms in cognitively normal older adults a better predictor of cognitive decline than either separately. Or is apathy, which has been related to neurofibrillary tangles in AD (Skogseth et al., 2008) when combined with elevated plasma t-tau a better predictor? To what extend do other neurobehavioral symptoms such as anxiety and worry along with elevated plasma t-tau predict the development of dementia? It would be useful to assess the relationship of the combination of elevated plasma t-tau and specific affective symptoms to different types of dementing disorders as it has been shown that different neuropsychiatric symptoms predict different subtypes of dementia (Liew, 2020). It may well be that any of these affective symptoms when combined with elevated plasma t-tau may be useful indicators of future cognitive decline due to the fact that the scores on the measures of affective symptoms used in this study are highly correlated. Additionally, all of these neurobehavioral symptoms can be viewed as stressors and chronic stress has been shown to exacerbate tau burden (Arenaza-Urquijo et al., 2020).

If future longitudinal research supports the utility of the combination of plasma t-tau and these potentially modifiable neurobehavioral syndromes in predicting the development of dementia, the assessment of plasma t-tau when patients present with complaints of depression, apathy, anxiety, worry or daytime sleepiness may be clinically relevant. Treating these affective symptoms of these patients may forestall later tau related cognitive decline.

## Author’s Note

The content of this article is solely the responsibility of the authors and does not necessarily represent the official views of the National Institutes of Health.

## Data Availability Statement

The raw data supporting the conclusions of this article will be made available by the authors, without undue reservation.

## Ethics Statement

The studies involving human participants were reviewed and approved by the University of North Texas Health Science Center IRB (IRB protocols 2016-128 and 2017-165) and is in accordance with Code of Ethics of the World Medical Association Declaration of Helsinki. The patients/participants provided their written informed consent to participate in this study.

## Author Contributions

JH was involved in designing the project, writing, and revising the manuscript. MP reviewed and made substantial edits to the manuscript. LJ was involved in reviewing and editing the manuscript. SO’B was involved designing the project and writing the manuscript. All authors made substantial contributions to the creation and writing of this manuscript, agree with the findings and have given consent to include their names on this manuscript.

## Conflict of Interest

SO’B has multiple patents on precision medicine for neurodegenerative diseases. The remaining authors declare that the research was conducted in the absence of any commercial or financial relationships that could be construed as a potential conflict of interest.

## Publisher’s Note

All claims expressed in this article are solely those of the authors and do not necessarily represent those of their affiliated organizations, or those of the publisher, the editors and the reviewers. Any product that may be evaluated in this article, or claim that may be made by its manufacturer, is not guaranteed or endorsed by the publisher.
